# Effect of gut microbiota from Henoch-Schönlein Purpura patients on acid-sensitive ion channel 3 expression and intestinal motility in germ-free rats

**DOI:** 10.1186/s12887-021-03013-3

**Published:** 2021-12-01

**Authors:** Jin-kun Wang, Bo Yan, Jun-mei Zhao, Li-ping Yuan

**Affiliations:** 1 Department of Pediatrics, First Affiliated Hospital of Anhui Medical University, Hefei, 230022 China; 2 Department of Medical Technology, Anhui Medical College, Hefei, 230026 China

**Keywords:** Gut motility, Gut microbiota, Henoch-Schönlein Purpura, Acid-sensing ion channel-3

## Abstract

**Background:**

It has been proven that gut microbiota alterations are involved in the development of Henoch-Schönlein Purpura (HSP). However, the pathogenesis of HSP hasn’t been eluciated. This study was to investigate the impact of gut microbiota from HSP on ASIC3 expression and interactions between microbiota and ASIC3 expression in the development of HSP.

**Methods:**

Feces collected from HSP and healthy children at the First Affiliated Hospital of Anhui Medical University were made into fecal microbial solutions. Germ-free rats were randomly assigned to either the control or HSP groups. The HSP group of rats were administered the fecal microbiota solution of HSP children, while the control group rats were administered the fecal microbiota solution of healthy children. Abdominal withdrawal reflex (AWR) and intestinal propulsion rate of the rats were used to determine visceral sensitivity. Composition of the gut microbiota of HSP children was determined using 16S rRNA gene sequencing. ASIC3 expression in the colon was ascertained through qRT-PCR as well as western blotting analysis.

**Results:**

The results showed a reduction in the number of species and abundance in the intestinal microbiota of children with HSP. Visceral sensitivity and intestinal propulsion rate of HSP group rats increased significantly, compared with the control group. Colon ASIC3 mRNA and protein levels in the HSP group were found to be upregulated. The microbiota dysbiosis of HSP patients could stimulate ASIC3 expression in the colon of Germ-free rats, which in turn affected intestinal motility.

**Conclusions:**

These results suggested that HSP children had intestinal microbiota disorder, which might affect gut motility by down-regulating colon ASIC3 expression in rats.

## Introduction

Henoch-Schönlein purpura (HSP), a condition in which small blood vessels are inflamed, is common in children and symptoms include a purpuric rash, gastrointestinal manifestations, arthritis and glomerulonephritis [1]. About 50–75% of children with HSP show gastrointestinal symptoms, such as GI bleeding and refractory abdominal pain. Complications, such as intestinal perforation, intussusception and intestinal obstruction, may accompany severe cases [2]. The early introduction of glucocorticoid may be beneficial for most HSP patients with gastrointestinal symptoms [3]. However, for some patients, steroid administration may not lead to remission. Increasing evidence has confirmed that acid sensing ion channel 3 (ASIC3) expression in the intrinsic enteric nervous system (ENS) acts as a pH sensor that is sensitive to mild extracellular acidification and altered ASIC3 levels mediate pain associated with tissue acidosis after inflammation [4–6]. Recent studies have found an association between HSP development and significant structural, as well as compositional gut microbial changes [7]. The resulting microbial dysbiosis may produce metabolites and short-chain fatty acids (SCFAs) that alter gastrointestinal tract pH. Therefore, we speculated that microbial dysbiosis affects ASIC3 expression in HSP patients and that these interactions may be involved in the development of HSP. This study investigated the impact of the gut microbiota from HSP on ASIC3 expression of Germ-free rats and interactions between the microbiota and ASIC3 expression for the development of HSP.

## Methods

### HSP children and healthy controls

Stool samples from 6 HSP children (3 females aged 4, 5 and 6 years old; 3 males aged 4, 5 and 6 years old) with severe abdominal pain and 6 healthy volunteers were collected, and mixed of six samples from the same group, and made into a solution of fecal bacteria [8]. The liquid was stored at − 80 °C in a refrigerator. HSP was diagnosed in the patients using EULAR/PReS endorsed consensus criteria [9]. The exclusion criteria used for these volunteers were as follows: (1) suffered from known metabolic or gastrointestinal diseases; (2) received an invasive medical intervention within the past 3 months; (3) used Antimicrobials (antibiotics, antifungals, antivirals) or probiotics that may alter fecal microbial composition up until 3 months before samples were taken; and (4) accepted any foods (eg, nuts) to which the recipient has a known food allergy.

First Affiliated Hospital of Anhui Medical University’s Clinical Research Ethics Committee provided approval for the protocol used in this clinical study (20200223). All participants/their guardians provided written informed consent.

### Preparation of rats for experiments

All animal experiments were performed in accordance with the“Animal Research: Reporting of In Vivo Experiments”(ARRIVE) guidelines.20 male Sprague-Dawley rats (4 weeks old, 90–120 g bw) were obtained from Anhui Medical University’s Medical Animal Laboratory. The rats were housed under 12 h light/dark cycles at 21 °C in an environmentally controlled room and were placed on wood chips in polycarbonate cages with positive-pressure sterile isolators. The rats were granted free access to sterilized drinking water and were fed an irradiated standard rodent diet (UAR, France).

Oral administration of 200 mg·kg-1 each of streptomycin, neomycin sulfate and bacitracin, following published methods [10], were used to create pseudo germ-free (GF) model rats. The pseudo GF rats were randomly assigned into either the healthy normal control (NC) group or the HSP group, using the random number table method. One milliliter of the settled suspension from HSP patients or normal health children, was administered daily to the pseudo GF rats for seven consecutive days. The Ethics Committee of Anhui Medical University (LLSC20200283) approved all experimental procedures, which complied with the National Institutes of Health Guidelines for the Care and Use of Laboratory Animals (NIH Publication No. 80–23).

### Colorectal distension and visceral sensitivity assessment of rats

A balloon was introduced into the colon of the rats and was inflated during colorectal distension (CRD) to induce a behavioral response. The intracolonic threshold necessary for a behavioral response was used as a measure of colonic hypersensitivity [11]. This response was observed through abdominal contraction and hind section elevation. The degree of colonic pain was evaluated using the abdominal withdrawal reflex (AWR) score. Flexible 2 cm latex balloons were ligated at the tip using a 2 mm catheter (Vygon, France) to create distention balloons. The rats were kept in the tunnel for 3 days before distention for acclimatization, to prevent the recording of artifacts due to movement and to keep the stress reaction at a minimum during distention. Deflated distention balloons were pushed into the descending colon of the rats so that its end was 1 cm proximal to the anus, after the rats were lightly anesthetized using isoflurane. In order to prevent displacement, the flexible catheter was fixed with tape to the base of the tail. After the procedure, 30 min of recovery time was provided before CRD initiation. An electronic barostat apparatus (Can Fu Mechanical and Electrical Co., Ltd., Shanghai, China) was used to conduct the CRD tests. The balloon was progressively inflated from 0 to 80 mmHg using 20 mmHg steps that lasted 5 min each. AWR scoring criteria: no behavioral response to balloon dilation stimulation, AWR 0; immobility of the rat body or occasionally clinches of the head during balloon dilation, AWR1; mild abdominal muscle contraction but without abdominal lifting, AWR2; abdominal muscle contraction and the abdomen lifted off the box platform, AWR3; body arching or lifting pelvic and scrotum off the platform, AWR4.

### Small intestinal transit experiment

After 24 h of fasting, each rat was administered with 2 ml of charcoal (10% gum arabic, 5% activated carbon). The rats were sacrificed after 30 min and the intestine from the pylorus to the ileocecal region was removed. Then, the total length from the pylorus to the ileocecal and the length from the pylorus to terminal carbon was measured after the intestine was straightened. The formula, intestinal propulsion rate (%) = advanced length of charcoal/total length of the small intestine × 100%, was used to calculate the rate of intestinal propulsion of each rat.

### Fecal DNA extraction and 16S rRNA gene sequencing

A QIAamp fast DNA stool Mini Kit (QIAGEN) was used to purify DNA extracted from the fecal samples [12]. The DNA purity and concentration were measured using agarose gel electrophoresis. Amplification of the variable regions, V3 and V4, of the bacterial 16S rRNA genes was performed using TransGen ap221–02 (TransGen) reagent, and sequenced on an Illumina MiSeq/HiSeq 2500 platform to generate pairedend reads.

High-throughput sequencing was performed to analyze the structure and diversity of fecal flora, as previously described [13]. Operational taxonomic units (OTUs) were clustered with 97% similarity, and taxonomic information was annotated using the Ribosomal Database Project (RDP) classifer. Four indices, Chao1, Observed species, Shannon and Simpson, which determined alpha diversity were used in QIIME (Version 1.8.0) to analyze species diversity.

### Quantitative real-time PCR (qRT-PCR)

Quantitative RT-PCR analysis was performed to analyze ASIC3 mRNA expression. Total RNA was extracted using Trizol reagent (Invitrogen, Australia). cDNA was produced using the GeneAmp RNA PCR kit. Gene expression was performed using the onestep RT-PCR kit with SYBR green in the ABI 7900 qPCR detection system. PCR reactions were performed with reverse transcription at 50 °C for 30 min, denaturation and reverse transcriptase in activation at 95 °C for 15 min, followed by 40 cycles (20 s each) of denaturation at 94 °C, and annealing and extension at 65 °C for 20 s. Melt curve analysis was performed to confirm the specificity of PCR products. PCR amplification data of each gene were normalized to Ct value of internal housekeeping gene (b-actin) from the same sample and the fold-changes in gene expression were calculated using the delta-delta Ct method.

The primers used were as follows: ASIC3 forward primer:5’AGGTCGGTGTGAACGGATTTG3’ and reverse primer:5’AGGTCGGTGTGAACGGATTTG3’; β-actin forward primer: 5’AGGTCGGTGTGAACGGATTTG3’ and reverse primer: 5’AGGTCGGTGTGAACGGATTTG3’.

### Western blotting analysis

The Bradford method was used to ascertain the protein concentrations of the protein lysates from the colon, which were prepared using a RIPA lysis buffer. After SDS-PAGE (8–12%) was performed on 50 μg of each protein sample, the sample was transferred onto a PVDF membrane and blocked with 5% non-fat milk in TBST for 1 h. Then, ASIC3 was used as the primary antibody to incubate the membranes at 4 °C, overnight. Finally, an HRP-conjugated secondary antibody was used to incubate the membranes for 60 min at 37 °C. An ECL luminescent detection system was used to detect luminescent signals and Image J software was used to analyze the intensity of the protein bands and β-actin expression was used for normalization.

### Statistical analysis

The results are presented as mean ± SD. GraphPad InStat (USA) and SPSS 17.0 (SPSS, USA) software were used to conduct all statistical analyses. Means were compared using the unpaired t-test and statistical significance was considered to be shown by *P* values of < 0.05.

## Results

### Visceral hypersensitivity and enhances intestinal motility

**Fig. 1:**
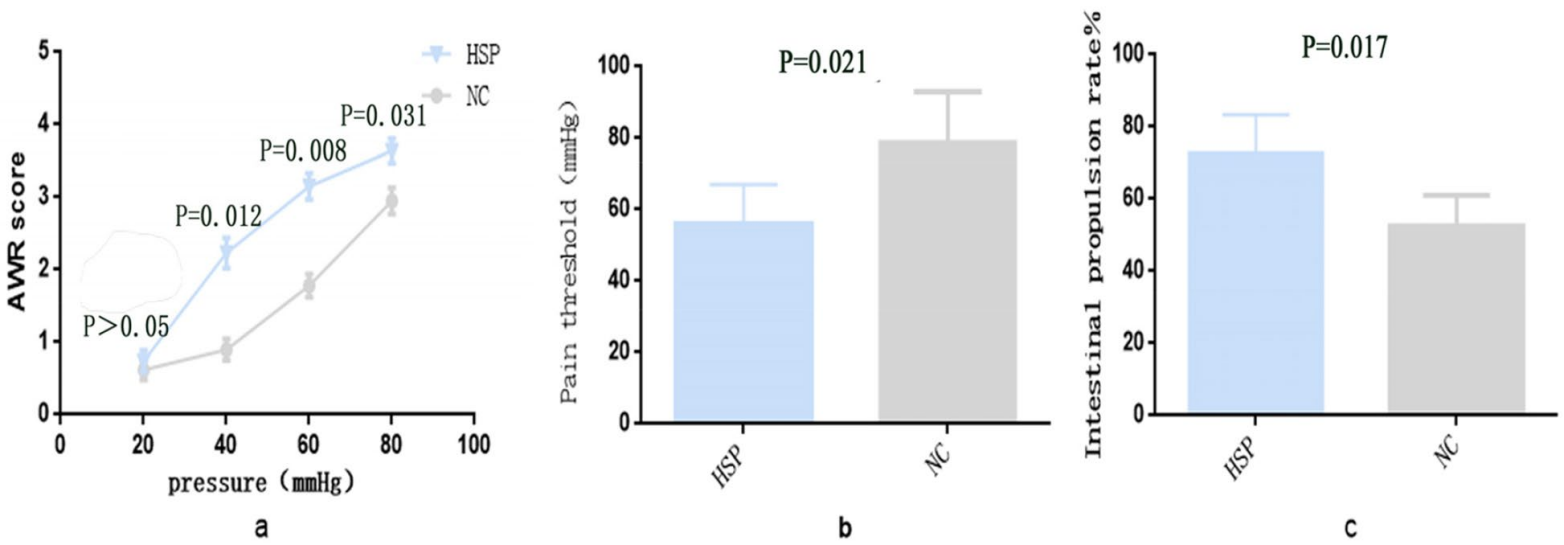
Visceral sensitivity and intestinal motility in rats. **a**. scores of AWR under different pressure of the colon balloon stimulation in CRD experiment; **b**. pain threshold of abdominal contractile reflex induced by balloon colorectal dilatation; **c**. intestinal propulsion rate, *n* = 10. HSP group: germ-free rats administered with the settled stool suspension from HSP patients. NC group: germ-free rats administered with the settled stool suspension from healthy children. Data were expressed as Mean ± Standard, *n* = 10

Visceral pain in rats is usually determined using CRD, due to robust reproducibility and ease of use. The pain threshold and score of AWR, which were assessed though observations of hind section elevation and abdominal contractions, were the parameters recorded. In our experiment, no significant differences were observed in AWR scores between the two groups at 20 mmHg pressure (P>0.05), while the AWR scores at 40, 60, 80 mmHg pressure in the HSP group of rats were significantly elevated, compared with that of rats in the control group (*P* = 0.012, *P* = 0.008, *P* = 0.031; Fig. 1a). The threshold of abdominal contractile reflex induced by balloon colorectal dilatation in HSP group rats was smaller than that of control group rats (*P* = 0.021; Fig. 1b). These findings showed that the microbiota of HSP children induced visceral hypersensitivity. Moreover we investigated the impact of the intestinal microbiota of HSP children on intestinal motility of rats and found that the intestinal propulsion rate of rats in the HSP group were significantly higher than that of control group rats (*P* = 0.017; Fig. 1c).

### Expression of colon ASIC3 at mRNA and protein levels in rats inoculated with fecal microbiota

**Fig. 2:**
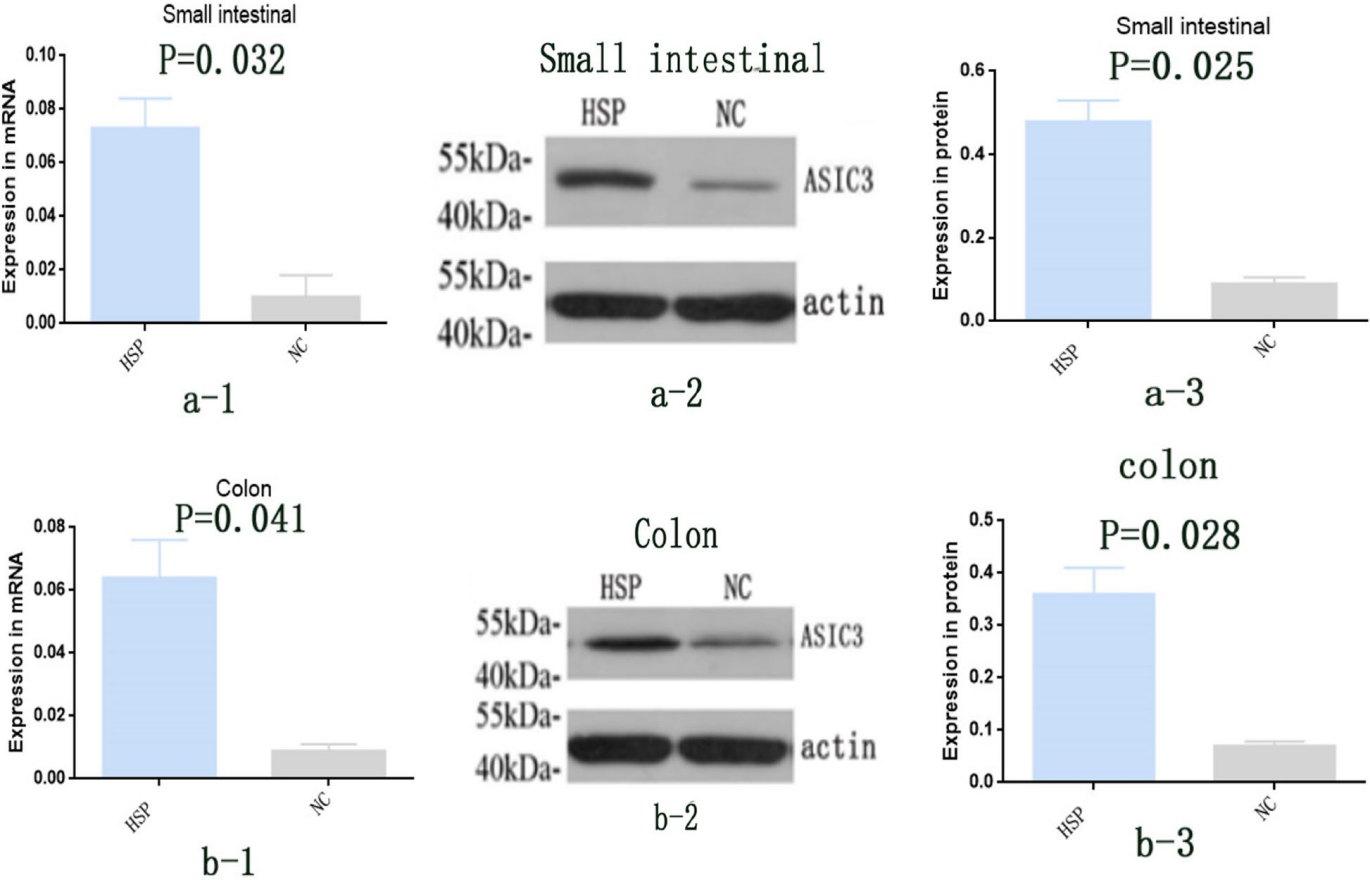
Expression of ASIC3 mRNA and protein in colon of rats, **a-1**.ASIC3 mRNA expression in small intestinal of rats (qRT-PCR); **a-2**: ASIC3 protein expression in small intestinal of rats (Western blot); a-3: ASIC3 protein expression in small intestinal of rats, respectively (Western blot), *n* = 10. **b-1**.ASIC3 mRNA expression in colon of rats (qRT-PCR); **b-2**: ASIC3 protein expression in colon of rats (Western blot); **b-3**: ASIC3 protein expression in colon of rats, respectively (Western blot), *n* = 10. HSP group: germ-free rats administered with the settled stool suspension from HSP patients. NC group: germ-free rats administered with the settled stool suspension from healthy children. Data were expressed as Mean ± Standard, *n* = 10

From each rat, 100 mg of small intestinal and colon tissue were obtained and qRT-PCR was used to determine ASIC3 mRNA expression. ASIC3 mRNA expression in the small intestinal and colon of HSP group rats was significantly elevated, compared with NC group rats (*P* = 0.032, 0.041; Fig. 2a-1, b-1).

**Fig. 3:**
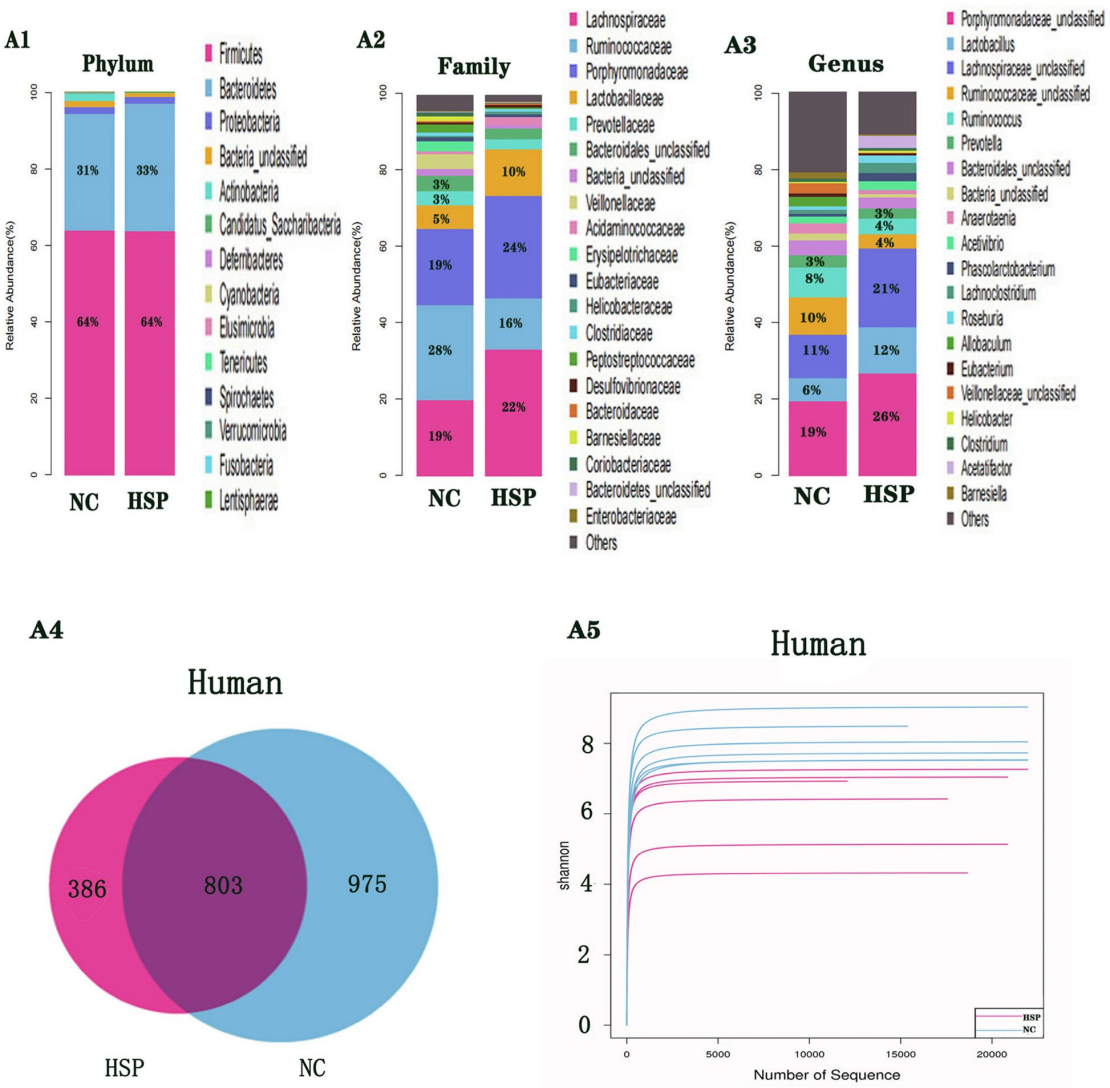
1 Composition of bacterial communities based 16S rDNA sequences in HSP patients, Normal Health Children **A1**. Intestinal flora composition at phylum level; **A2**. Intestinal flora composition at family level; **A3**. Intestinal flora composition at genus level. Data were expressed as Mean ± Standard *n* = 6. 2 Overlapping Venn diagram of OTUs and Shannon exponential curve in HSP patients, Normal Healthy Children OTUs = operational taxonomic units. Shannon exponential curve was used to analyze species abundance and species evenness. **A4**. Overlapping Venn diagram of OTUs; **A5**. Shannon exponential curve. Data were expressed as Mean ± Standard *n* = 6

Proteins were extracted from the small intestinal and colon tissue and western blotting analysis was used to determine ASIC3 protein expression. ASIC3 protein expression in the small intestinal and colon of HSP group rats was significantly elevated, compared with NC group rats (*P* = 0.025, *P* = 0.028; Fig. 2 a-2, 2b-2; Fig. 2a-3, b-3).

### Intestinal microbial analysis of HSP and healthy children

16S rRNA gene sequencing was performed on stool obtained from HSP children and healthy children to investigate the composition of their intestinal microbiota. The results showed that HSP children and healthy children had different compositions of intestinal microbiota. The dominant bacteria of HSP children and healthy children at the phylum level were firmicutes, bacteroidetes and proteobacteria, and the most dominant bacteria was firmicutes **(**Fig. 3-1, A1). Lachnospiraceae, ruminococcaceae, porphyromonadaceae and lactobacillaceae were the dominant bacteria of HSP children and healthy children at the family level. However, porphyromonadaceae was the most dominant family of bacteria in HSP children (24%), while ruminococcaceae was the most dominant family of bacteria in healthy children (28%)(Fig. 3-2, A2). At the genus level, the dominant bacteria of healthy children were porphyromonadaceae, lachnospiraceae and ruminococcaceae (19, 11, 10% respectively), the dominant bacteria of HSP children were porphyromonadaceae, lachnospiraceae and lactobacillus (26, 21, 12% respectively)(Fig. 3-1**,** A3).

386 OTUs were identified in HSP patients, while 975 OTUs were identified in healthy controls, of which 803 were common to both groups. The difference in OTUs between the groups was significant (*P* = 0.023) and is shown in Fig. 3-2 A4 and Table 1.

**Table 1:** Analysis of α diversity index between HSP patients and normal healthy children (χ ± S, *n* = 6)

Group	OTU	α diversity			
		Chao1	Observesd-species	Shannon	Simpson
**HSP**	**198土65**	**415.69土123**	**243.17土65**	**3.94土0.62**	**0.46土0.06**
**NC**	**296土73**	**395.65土97**	**216.74土59**	**5.78土0.51**	**0.89土0.13**
***t***	**2.53**	**3.25**	**2.41**	**8.56**	**2.21**
***p***	**0.023**	**0.217**	**0.089**	**0.022**	**0.008**

*OTUs* Operational taxonomic units

The diversity indices (Chao1, Observed-species, Shannon, Simpson index) of the samples were determined in this study. Shannon exponential curves indicated that the sequencing data was large enough to reflect microbial information of each group when the curve tended to be flat due to the increase in sequencing depth, as shown in Fig. 3-2 A5. The indices, Chao1 and observed-species, which were used to describe the number of species were found to be slightly elevated in HSP patients, compared with the controls, with no significant differences found between them. Compared with healthy controls, the Shannon and Simpson indices were significantly smaller in HSP children (*P = 0.008, P = 0.022*). These results are shown in Table 1.

## Discussion

The intestinal microbiota is highly involved in maintaining the intestinal environment, the occurrence and self-stabilization of intestinal diseases, and factors that cause a loss of balance and stability result in the occurrence of many diseases [14]. Mounting evidence has conveyed that alterations of the intestinal flora occur during Henoch-Schönlein Purpura (HSP) development [7, 15, 16] The present study demonstrated that there was a difference in the bacterial composition and relative abundance between HSP patients and controls. OTU abundance and indices of intestinal microbiota diversity (Shannon and Simpson) were significantly lower in HSP patients, compared with controls, which suggested that species composition and abundance of intestinal microbiota in patients with abdominal HSP had been obviously altered.

Under normal conditions, the majority of the small intestine is aerobic with a pH of 5.5 to 7.0 and contains gram-negative bacteria. The colon has higher numbers of anaerobic bacteria and the dominant bacteria are Bacteroides, Bifidobacterium and fungi, while its pH ranges from 6.1 to 7.5. These intestinal florae are used to produce vitamins, fatty acids, bile acids and other products, while propionic acid, butyric acid, acetic acid and other short chain fatty acids (SCFAs) are usually the fermentation products of anaerobic bacteria [17, 18]. The presence of a large number of SCFAs can reduce intestinal pH [19].

Alterations to the intestinal microbiota could trigger or aggravate visceral hypersensitivity in functional gastrointestinal disorder diseases and affect gastrointestinal motility [20–23]. Studies have shown that intestinal bacteria may participate in the pathophysiological process of HSP, and the microbiota may be highly involved in the occurrence of abdominal pain in HSP patients [24]. In this study, AWR scores and intestinal propulsion rates were found to be significantly increased and the pain threshold of the abdominal contractile reflex was obviously decreased in rats of the HSP group, compared with rats in the NC group. These results suggested that the intestinal microbiota of HSP patients could induce visceral pain and prompt GI motility of Germ-free rats. However, knowledge on the distinctive molecular mechanism by which intestinal flora are involved is not clear.

Moreover, the imbalance in intestinal microbiota in functional gastrointestinal disorders (FGIDs) has been proven to increase the level of intestinal endotoxins and the production of pro-inflammatory factors as a result of damage caused to the intestinal mucosal barrier and changes in intestinal permeability, which lead to chronic low-grade inflammation and a decrease in the pH of the intestinal environment [19, 25]. In our experiments, no differences in the bacterial phyla were found between HSP patients and healthy children, but significant changes in composition and structure were detected between gut microbial families of HSP patients and controls. Moreover, the higher relative abundance of the Lachnospiraceae, Porphyromonadacea and Lactobacilli families were found in HSP patients, compared with controls. The dominant types of bacteria found in HSP patients are involved in the production of SCFAs [26]. Therefore, more SCFAs were produced in the intestinal tract of HSP patients, which decreased the pH of the intestinal environment. ASIC channels have been implicated in many pathophysiological and physiological processes related to extracellular pH variations [27]. ASIC3 is a major ASIC subunit that is predominantly expressed in the nervous system and also in areas of the non-nervous system. It is involved in the mediation of various pain qualities during inflammation, ischemia or cancer metastases, which involve a decrease in the pH of the cellular interstitium of cutaneous or muscle tissue [28, 29]. Research studies have shown that ASIC3 is highly involved in the decrease of the pain threshold in an inflammatory environment and in the development of esophageal hypersensitivity [30]. Our study showed that the mRNA and protein expressions of ASIC3 in colon were significantly elevated in rats of the HSP group, compared with that of rats in the NC group. This suggested that intestinal microbiota in patients with abdominal HSP stimulated ASIC3 expression in the colon of Germ-free rats.

Researches proved that some acid inhibitors such as cimetidine could improve the the abdominal pain and purpura of HSP children [31, 32]. So we speculate that intestinal microbiota might increase the intestinal motility by stimulating ASIC3 expression in colon, which will be further studied in our future. The present findings showed that alterations in the intestinal microbiota in HSP patients was closely related to GI dysmotility and ASIC3 expression of Germ-free rats. And ASIC3 expression and open might be involved in the GI disorder mediated by intestinal flora alterations in HSP patients. The results of this study will enhance our understanding for the mechanism of HSP and identification of potential therapies that may be used to treat and prevent HSP in children.

## Conclusions

Our data firstly find that intestinal microbiota in HSP patients can stimulate ASICs expression and GI motility of Germ-free rats. However, the underlying mechanisms in this study remain unclear, whether ASICs is involved in the mechanism of microbiota regulating intestinal motility requires further investigation. Future research should focus on exploring the therapeutic effect of fecal microbiota transplantation (FMT) on Henoch-Schönlein Purpura patients, identifying specific microbiota that play a key role in the development of HSP and clarifying their functions, so as to accurately manipulate gut microbiota to improve the quality of life of HSP patients. Our better understanding of microbiota-gut interactions might provide new targeted therapies for HSP patients, which will be further performed in our study.

## Data Availability

The datasets used and/or analysed during the current study are available from the corresponding author on reasonable request.

## Abbreviations

ASIC3: Acid sensing ion channel3
AWR: Abdominal withdrawal reflex
CRD: colorectal distension
ENS: enteric nervous system
FGIDs: functional gastrointestinal disorders
GI: Gastrointestinal
HSP: Henoch-Schönlein Purpura
SCFAs: short chain fatty acids

## References

[1] Trnka P (2013). Henoch-Schönlein purpura in children. J Paediatr Child Health.

[2] Zhang Y, Huang X (2008). Gastrointestinal involvement in Henoch–Schönlein purpura. Scand J Gastroenterol.

[3] Wang H, Zhang B, Li S, Ou R, Liu Y, Tan W (2020). Clinical outcome in pediatric refractory gastrointestinal Henoch-Schönlein purpura treated with mycophenolate mofetil. Eur J Pediatr.

[4] Wang X, Zhang L, Wang Y, Liu X, Zhang H, Liu Y, Shen N, Yang J, Gai Z (2018). Gut microbiota dysbiosis is associated with Henoch-Schönlein Purpura in children. Int Immunopharmacol.

[5] Xu S, Tu W, Wen J, Zhou H, Chen X, Zhao G, Jiang Q (2014). The selective ASIC3 inhibitor APETx2 alleviates gastric mucosal lesion in the rat. Pharmazie.

[6] Scalera A, Loguercio C (2012). Focus on irritable bowel syndrome. Eur Rev Med Pharmacol Sci.

[7] Holzer P (2015). Acid-sensing ion channels in gastrointestinal function. Neuropharmacology.

[8] Crouzet L, Gaultier E, Del'Homme C, Cartier C, Delmas E, Dapoigny M, Fioramonti J, Bernalier-Donadile A (2013). The hypersensitivity to colonic distension of IBS patients can be transferred to rats through their fecal microbiota. Neurogastroenterol Motil.

[9] Ozen S, Ruperto N, Dillon MJ, Bagga A, Barron K, Davin JC, Kawasaki T, Lindsley C, Petty RE, Prieur AM, Ravelli A (2006). Woo P.EULAR/PReS endorsed consensus criteria for the classification of childhood vasculitides. Ann Rheum Dis.

[10] Bhowmik SK, An JH, Lee SH, Jung BH (2012). Alteration of bile acid metabolism in pseudo germ-free rats. Arch Pharm Res.

[11] Spence MJ, Moss-Morris R (2007). The cognitive behavioural model of irritable bowel syndrome: a prospective investigation of patients with gastroenteritis. Gut.

[12] Feng Y, Zhang X, Su L, Zhang Y, He F (2019). A supersensitive MSPQC bacterium sensor based on 16S rRNA and “DNA-RNA switch”. Biosens Bioelectron.

[13] Vezzulli L, Stagnaro L, Grande C, Tassistro G, Canesi L, Pruzzo C (2018). Comparative 16SrDNA gene-based microbiota profiles of the Pacific oyster (Crassostrea gigas) and the Mediterranean mussel (Mytilus galloprovincialis) from a shellfish farm (Ligurian Sea, Italy). Microb Ecol.

[14] Saffouri GB, Shields-Cutler RR, Chen J, Yang Y, Lekatz HR, Hale VL, Cho JM, Battaglioli EJ, Bhattarai Y, Thompson KJ, Kalari KK, Behera G, Berry JC, Peters SA, Patel R, Schuetz AN, Faith JJ, Camilleri M, Sonnenburg JL, Farrugia G, Swann JR, Grover M, Knights D, Kashyap PC (2019). Small intestinal microbial dysbiosis underlies symptoms associated with functional gastrointestinal disorders. Nat Commun.

[15] Chen B, Wang J, Wang Y, Zhang J, Zhao C, Shen N, Yang J, Gai Z, Zhang L (2018). Oral microbiota dysbiosis and its association with Henoch-Schönlein Purpura in children. Int Immunopharmacol.

[16] Zhang Y, Xia G, Nie X, Zeng Y, Chen Y, Qian Y, Chen G, Huang J, Wang C, Zhang C, Huang X, Yang Y, Qiu X, Yang F, Chen J, Hu J (2021). Differences in manifestations and gut microbiota composition between patients with different Henoch-Schonlein Purpura phenotypes. Front Cell Infect Microbiol.

[17] Simrén M (2018). Manipulating the gut microbiome as a treatment strategy for functional gastrointestinal disorders. Gastroenterology.

[18] Galloway-Peña JR, Peterson CB, Malik F, Sahasrabhojane PV, Shah DP, Brumlow CE, Carlin LG, Chemaly RF, Im JS, Rondon G, Felix E, Veillon L, Lorenzi PL, Alousi AM, Jenq RR, Kontoyiannis DP, Shpall EJ, Shelburne SA, Okhuysen PC (2019). Fecal Microbiome, Metabolites, and Stem Cell Transplant Outcomes: A Single-Center Pilot Study. Open Forum Infect Dis.

[19] Nicolas GR, Chang PV (2019). Deciphering the chemical lexicon of host-gut microbiota interactions. Trends Pharmacol Sci.

[20] Hyman PE, Milla PJ, Benninga MA, Davidson GP, Fleisher DF, Jan T (2016). Childhood functional gastrointestinal disorders: neonate/toddler. Gastroenterology.

[21] Lloyd-Price J, Arze C, Ananthakrishnan AN, Schirmer M, Avila-Pacheco J, Poon TW, Andrews E, Ajami NJ, Bonham KS, Brislawn CJ, Casero D, Courtney H, Gonzalez A, Graeber TG, Hall AB, Lake K, Landers CJ, Mallick H, Plichta DR, Prasad M, Rahnavard G, Sauk J, Shungin D, Vázquez-Baeza Y, Richard A, Braun J, Denson LA, Jansson JK, Knight R, Kugathasan S, DPB MG, Petrosino JF, Stappenbeck TS, Winter HS, Clish CB, Franzosa EA, Vlamakis H, Xavier RJ, Huttenhower C, White III, IBDMDB Investigators (2019). Multi-omics of the gut microbial ecosystem in inflammatory bowel diseases. Nature.

[22] Labus JS, Osadchiy V, Hsiao EY, Tap J, Derrien M, Gupta A, Tillisch K, Nevé BL, Grinsvall C, Ljungberg M, Öhman L, Törnblom H, Simren M, Mayer EA (2019). Evidence for an association of gut microbial Clostridia with brain functional connectivity and gastrointestinal sensorimotor function in patients with irritable bowel syndrome, based on tripartite network analysis. Microbiome.

[23] He C, Wang H, Liao WD, Peng C, Shu X, Zhu X, Zhu XH (2019). Characteristics of mucosa-associated gut microbiota during treatment in Crohn's disease. World J Gastroenterol.

[24] Saps M, Dhroove G, Chogle A (2011). Henoch-Schonlein purpura leads to functional gastrointestinal disorders. Dig Dis Sci.

[25] Haiwen Z, Rui H, Bingxi Z, Guan QF, Zeng JF, Wang XM, Wang BB (2019). Oral Administration of Bovine Lactoferrin-Derived Lactoferricin (Lfcin) B could attenuate Enterohemorrhagic Escherichia coli O157:H7 induced intestinal disease through improving intestinal barrier function and microbiota. J Agric Food Chem.

[26] Strain CR, Collins KC, Naughton V, McSorley EM, Stanton C, Smyth TJ, Soler-Vila A, Rea MC, Ross PR, Cherry P, Allsopp PJ (2020). Effects of a polysaccharide-rich extract derived from Irish-sourced Laminaria digitata on the composition and metabolic activity of the human gut microbiota using an in vitro colonic model. Eur J Nutr.

[27] Immke DC, Mccleskey EW (2001). ASIC3: a lactic acid sensor for cardiac pain. Sci World J.

[28] Alvarez de la Rosa D, Zhang P, Shao D, White F, Canessa CM (2002). Functional implications of the localization and activity of acid-sensitive channels in rat peripheral nervous system. Proc Natl Acad Sci U S A.

[29] Lin SH, Cheng YR, Banks RW, Min MY, Bewick GS, Chen CC (2016). Evidence for the involvement of ASIC3 in sensory mechanotransduction in proprioceptors. Nat Commun.

[30] Wu L, Oshima T, Shan J, Sei H, Tomita T, Ohda Y, Fukui H, Watari J (2015). Miwa H.PAR-2 activation enhances weak acid-induced ATP release through TRPV1 and ASIC sensitization in human esophageal epithelial cells. Am J Physiol Gastrointest Liver Physiol.

[31] Zhang F, Chen L, Shang S, Jiang K (2018). Atypical purpura location in a pediatric patient with Henoch-Schönlein purpura: a case report. Medicine (Baltimore).

[32] Liang J, Yang HY, Yang QZ (2007). Ligustrazine and cimetidine therapy for the prevention of Henoch-Schonlein purpura nephritis in children: a follow-up study of 380 cases. Zhongguo Dang Dai Er Ke Za Zhi.

